# High DNA Uptake Capacity of International Clone II *Acinetobacter baumannii* Detected by a Novel Planktonic Natural Transformation Assay

**DOI:** 10.3389/fmicb.2019.02165

**Published:** 2019-09-24

**Authors:** Yuan Hu, Lihua He, Xiaoxia Tao, Fanliang Meng, Jianzhong Zhang

**Affiliations:** State Key Laboratory of Infectious Disease Prevention and Control, Collaborative Innovation Center for Diagnosis and Treatment of Infectious Diseases, National Institute for Communicable Disease Control and Prevention, Chinese Center for Disease Control and Prevention, Beijing, China

**Keywords:** *Acinetobacter baumannii*, natural transformation, competence induction, international clonal lineage 2, DNA uptake

## Abstract

Acquisition of novel resistance genes is a key driver of multidrug resistance in the nosocomial pathogen *Acinetobacter baumannii*. To investigate the DNA uptake ability among clinical *A. baumannii* strains, a planktonic salt-free transformation assay was developed. A total of 142 clinical *A. baumannii* isolates with divergent genetic distance were selected, and 86 of them belong to international clonal lineage II (ICL2). Using this new transformation assay, 38% of the clinical *A. baumannii* isolates were natural competent. Among the multidrug-resistant (MDR) isolates, the transformable isolates all belonging to the ICLs, and showed significant higher transformation frequency compared with sensitive isolates. In addition, some of the ICL2 isolates triggered competence much earlier than the sensitive isolates with similar transformation frequencies. This may give them more opportunities to obtain successful transformation in their natural environment and provides an important clue to explain the severe drug resistance and clinical successfulness of ICL2.

## Introduction

*Acinetobacter baumannii* is an important opportunistic pathogen that has caused enormous public health concerns worldwide because of its remarkable ability to develop antibiotic resistance (Howard et al., 2012). The population structure of *A. baumannii* isolates is diverse; for example, more than 600 multilocus sequence typing (MLST) sequence types (STs) are currently listed in the *A. baumannii* MLST database^1^, but only a few specific clones (international clonal lineages, ICLs) are distributed worldwide (Karah et al., 2012). ICL2, identified by the reference Pasteur’s MLST scheme as ST2, is by far the predominant and most widely distributed *A. baumannii* clone (Diancourt et al., 2010). Isolates from ICL2 have repeatedly shown high resistance rates to nearly all antimicrobial agents (Diancourt et al., 2010). The frequent acquisition of diverse antimicrobial resistance determinants is probably a major factor in the dissemination and worldwide spread of ICL2 isolates (Mugnier et al., 2010; Sahl et al., 2011). Thus, horizontal gene transfer events considerably contribute to the alarming resistance development of this emerging pathogen (Snitkin et al., 2011). Among the horizontal gene transfer mechanisms, natural transformation provides a convenient route for genetic exchanges and has been demonstrated in some *A. baumannii* isolates (Maria Soledad et al., 2010; Harding et al., 2013; Wilharm et al., 2013; Traglia et al., 2016; Godeux et al., 2018; Domingues et al., 2019). Most of the sequenced *Acinetobacter* plasmids do not harbor the genes required for conjugative transfer, so it proposed that natural transformation may also play an important role in plasmid transfer for *A. baumannii* (Fondi et al., 2010).

Natural transformation is a multistep process involving induction of natural competence state in which prokaryotes are able to take up genetic material from their surroundings, and the consequent uptake of DNA, processing of DNA, and its subsequent recombination into the chromosome or reconstitution of the plasmid DNA (Chen and Dubnau, 2004). As a new bacterial species capable of natural transformation, it is still unknown whether differences in natural transformation ability between *A. baumannii* strains could lead to drug resistance differences. To clarify this, we aimed to investigate the natural competence induction and DNA uptake ability of clinical *A. baumannii* isolates with different drug resistant levels.

The most well-studied transformable species in *Acinetobacter*, *Acinetobacter baylyi* strain ADP1, is always transformable during planktonic growth (Cruze et al., 1979; Averhoff and Friedrich, 2003). Unlike it, the vast majority of competent *A. baumannii* strains were detected by moving along wet surfaces (Wilharm et al., 2013, 2017; Godeux et al., 2018). However, this method is laborious when a large number of strains need to be tested. We describe a novel planktonic salt-free transformation assay for *A. baumannii* and significant variation in transformation frequency between multidrug-resistant (MDR) and sensitive isolates was revealed. Moreover, some isolates from ICL2 showed more efficient intrinsic transformation ability compared with the sensitive strains.

## Materials and Methods

### Bacterial Strains

The clonal relationships between *A. baumannii* isolates were assessed using pulsed-field gel electrophoresis (PFGE), as previously described (Harald et al., 2005). Similarity thresholds of 86% were arbitrarily used to define isolates belonging to the same PFGE cluster. MLST was performed according to the published Pasteur protocols (Diancourt et al., 2010). According to the PFGE profiles, a total of 142 clinical strains isolated from 18 hospitals (no more than 3 isolates of each PFGE type) were selected for this study.

The antimicrobial susceptibilities of all the isolates above were examined by *E* test on Mueller-Hinton agar, and the results were evaluated using the CLSI breakpoints for microdilution methods (Ferraro, 2010). A strain resistant to at least three classes of antimicrobial agents – all penicillins and cephalosporins (including inhibitor combinations), fluoroquinolones, and aminoglycosides – was called MDR (Manchanda et al., 2010).

### Construction of the Donor DNA Plasmid

The donor DNA plasmid pOri was constructed by cloning a PCR product of the replication origin region of pWH1266, which is sufficient for replication and stable maintenance of plasmids in *Acinetobacter*, into pCR-Blunt II-TOPO (Hunger et al., 1990). The zeocin resistance cassette of pCR-Blunt II-TOPO was used as the selectable marker for MDR *A. baumannii* (Luna et al., 2017).

### Transformation Assay

An overnight planktonic culture, started from a frozen stock, was diluted 1:200 into 200 μl fresh Luria-Bertani broth without sodium chloride (LB-no salt) containing 500 ng of donor DNA. After 4 h of incubation at 37° C with shaking, transformable cells were selected by LB-no salt agar with 250 μg/ml zeocin. Transformation frequencies were obtained by calculating the ratio of transformant CFUs (zeocin resistant) to the total number of CFUs evaluated according to OD600. The bacterial counts of CFU/ml/OD600 of bacteria were determined by plating serial dilutions of the cultures on LB agar.

To test the effect of different conditions, assays were performed by altering one parameter at a time. The temperature was switched to 28, 37, or 42°C. Different media were assayed, including LB, LB-no salt, tryptic soy broth (TSB), and nutrient broth (NB). The effects of sucrose (0, 20, 40, 80, 160, and 320 mM), sodium chloride (0, 0.2, 0.4, 0.6, 0.8, and 1%), KCl (0.64%), NaHCO_3_ (0.72%), and KH_2_PO_4_ (1.25%) were tested. All experiments were performed in triplicate, and statistical analysis was performed by SPSS24.

### Time Course Experiments

To follow the timing of competence development during growth in LB-no salt broth, samples (100 μl) were removed from the transformation assays at different time points (every 20 min until 4 h) and detected by zeocin selective agar.

## Results

### Factors Affecting the Natural Transformation of *A. baumannii*

**FIGURE 1:**
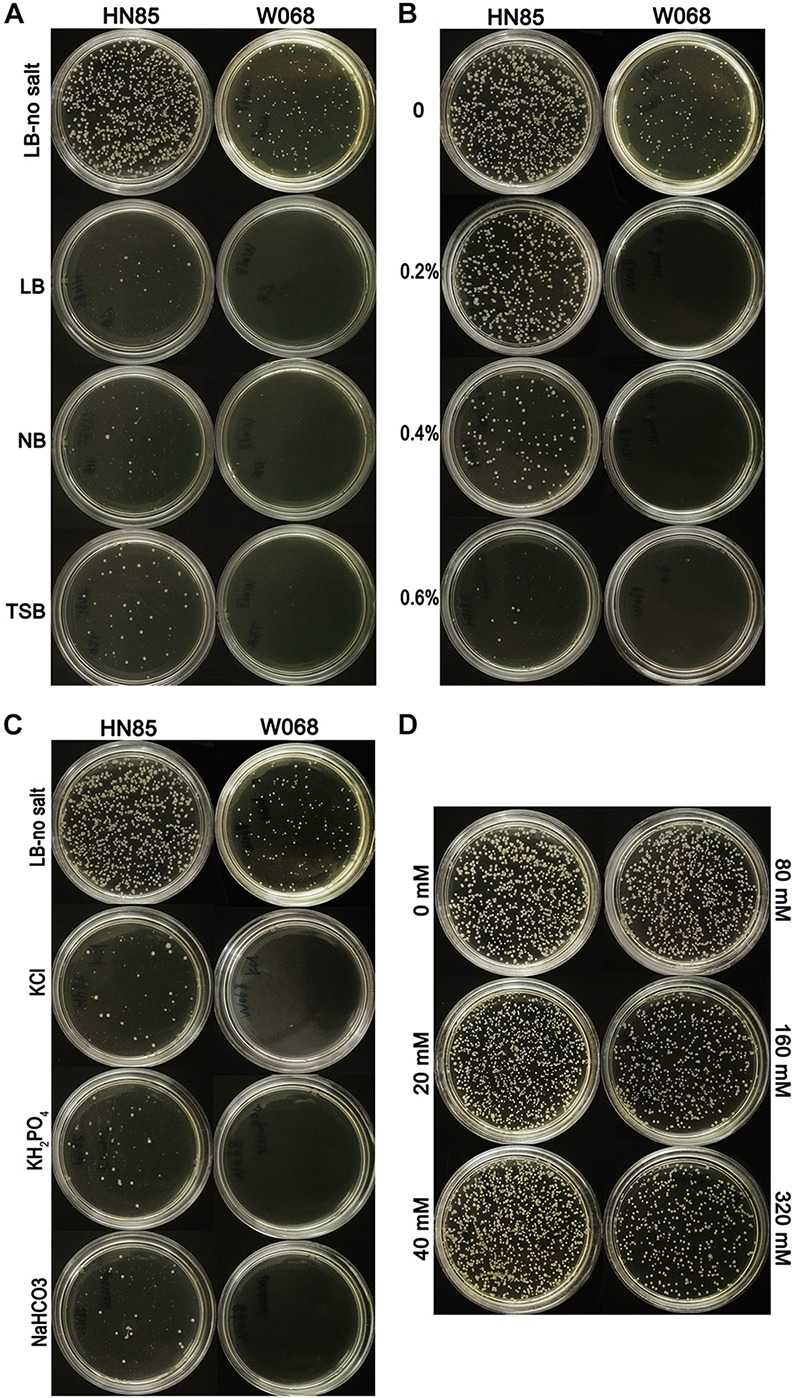
Transformability of *Acinetobacter baumannii* cells under different conditions. **(A)** Transformation assays with LB-no salt, LB, NB, and TSB with strains HN85 and W068. **(B)** Transformability of *A. baumannii* strain W068 and HN85 in LB-no salt broth with the addition of different concentrations of sodium chloride (0, 0.2 0.4, and 0.6%, wt/vol). **(C)** Transformability of *A. baumannii* strain HN85 and W068 in LB-no salt broth and LB-no salt with the addition of KCl (0.64%), NaHCO_3_ (0.72%), and KH_2_PO_4_ (1.25%). **(D)** Transformability of *A. baumannii* strain HN85 in LB-no salt broth with the addition of different concentrations of sucrose (0, 20, 40, 80, 160, and 320 mM).

The constructed donor DNA pOri was stable in *A. baumannii* and allowed for selection of the successful transformants in MDR isolates, which was confirmed by an electroporation test and the subsequent plasmid extraction. We challenged the natural transformation with four media, and only LB-no salt broth showed high transformation frequency and was selected for use in the subsequent transformation assay (Figure 1A). Successful pOri uptake was verified by PCR with the M13 primers for each tested strains. To avoid false positives yield by the remains of the donor DNA on the selected plate, all the checked colonies were transferred to a fresh selective plate before testing by colony PCR. The only difference between LB-no salt and LB broth is 1% NaCl in the latter, and the former is the only medium tested in this study with no NaCl. Therefore, we speculate that NaCl may have a negative effect on *A. baumannii* transformation.

The effect of NaCl was measured in 21 representative *A. baumannii* isolates. Higher salt concentrations drastically reduced the DNA uptake for all of the strains (Figure 1B), but the highest salt concentration at which transformants are still observed varied depending on the isolates (tolerance to NaCl, Table 1). The transformation frequency was also drastically reduced with KCl, NaHCO_3_ and KH_2_PO_4_ with molar concentration equivalent to 0.5% NaCl (Figure 1C), so the inhibition effect was not specific salt ion-dependent. In contrast, variations of the sucrose concentration showed no significant differences in transformation frequencies (Figure 1D), suggesting that the osmolarity of culture media does not influence *A. baumannii* transformation.

**TABLE 1:** Variations in NaCl tolerance between 21 representative *Acinetobacter baumannii* isolates.

**Strain**	**Drug resistance**	**MLST^a^**	**Mean transformation frequency in LB-no salt ± SD**	**NaCl tolerance^b^(%)**
S12471	S	ST240	3.94 × 10^–6^ ± 3.29 × 10^–6^	0.4
S13160	S	N5	3.22 × 10^–6^ ± 1.22 × 10^–6^	0.4
S12920	S	ST40	3.40 × 10^–6^ ± 1.21 × 10^–6^	0
W068	S	ST338	1.48 × 10^–6^ ± 4.62 × 10^–7^	0
S11013	S	ST40	8.26 × 10^–7^ ± 4.21 × 10^–7^	0
C249	S	N2	1.53 × 10^–7^ ± 1.21 × 10^–7^	0
C536	S	ST452	7.54 × 10^–8^ ± 0.84 × 10^–8^	0
HN085	MDR	ST2	3.24 × 10^–5^ ± 1.50 × 10^–5^	0.6
HN027	MDR	ST2	5.25 × 10^–6^ ± 2.00 × 10^–6^	0.2
R11	MDR	ST2	4.98 × 10^–6^ ± 3.98 × 10^–7^	0.8
BJ36	MDR	ST2	2.51 × 10^–6^ ± 1.50 × 10^–6^	0.4
FZ8	MDR	ST2	3.59 × 10^–5^ ± 2.28 × 10^–5^	0.6
FZ4	MDR	ST2	3.23 × 10^–5^± 2.42 × 10^–5^	0.4
R4	MDR	ST2	1.20 × 10^–5^ ± 2.50 × 10^–6^	0.2
R10	MDR	ST2	9.53 × 10^–6^ ± 4.20 × 10^–6^	0.8
HN2	MDR	ST2	8.22 × 10^–6^ ± 4.15 × 10^–6^	0.2
BJ46	MDR	ST2	2.24 × 10^–6^ ± 1.11 × 10^–6^	0.8
RM35	MDR	ST2	2.41 × 10^–6^ ± 2.10 × 10^–6^	0
A605	MDR	ST1	9.33 × 10^–6^ ± 7.48 × 10^–6^	0.8
A608	MDR	ST1	3.04 × 10^–6^ ± 2.71 × 10^–6^	0.4
A614	MDR	N10	2.59 × 10^–5^ ± 2.42 × 10^–5^	1.0

*^*a*^New STs revealed in this study were named with Arabic numerals beginning with the letter N. ^*b*^The highest salt concentration at which transformants are still observed.*

The effect of temperature was determined by using 16 representative strains. No significant difference in transformation frequencies was revealed between 27 and 37°C (Wilcoxon test, *P* = 0.836) (Table 2), but the transformation was drastically reduced at 42°C (Wilcoxon test, *P* < 0.01), indicating that *A. baumannii* transformation was not influenced by the common environment or human body temperature but was drastically reduced by extremely high temperatures.

**TABLE 2:** Mean transformation frequencies of *A. baumannii* strains at varying temperatures (27, 37, and 42°C).

**Strain**	**27°C**	**37°C**	**42°C**
A608	1.39 × 10^–5^	3.04 × 10^–6^	1.73 × 10^–7^
RM35	2.51 × 10^–6^	2.41 × 10^–6^	0
RM8	1.21 × 10^–6^	7.69 × 10^–7^	3.46 × 10^–8^
BJ46	5.97 × 10^–6^	2.24 × 10^–6^	2.41 × 10^–7^
BJ36	1.23 × 10^–5^	2.51 × 10^–6^	4.79 × 10^–8^
A605	2.21 × 10^–5^	9.33 × 10^–6^	1.09 × 10^–7^
FZ4	7.14 × 10^–6^	3.23 × 10^–5^	3.71 × 10^–7^
R10	2.82 × 10^–5^	9.53 × 10^–5^	7.36 × 10^–7^
S12184	1.23 × 10^–7^	2.50 × 10^–8^	0
S13160	1.42 × 10^–5^	3.22 × 10^–6^	0
HN085	1.35 × 10^–6^	3.24 × 10^–5^	3.82 × 10^–7^
S12920	3.76 × 10^–7^	3.40 × 10^–6^	0
W068	2.41 × 10^–7^	1.48 × 10^–6^	0
C536	7.67 × 10^–8^	7.54 × 10^–8^	0
S12471	1.72 × 10^–6^	3.94 × 10^–6^	0
C249	1.83 × 10^–7^	1.53 × 10^–7^	0

### Natural Transformation in Clinical *A. baumannii* Isolates

**FIGURE 2:**
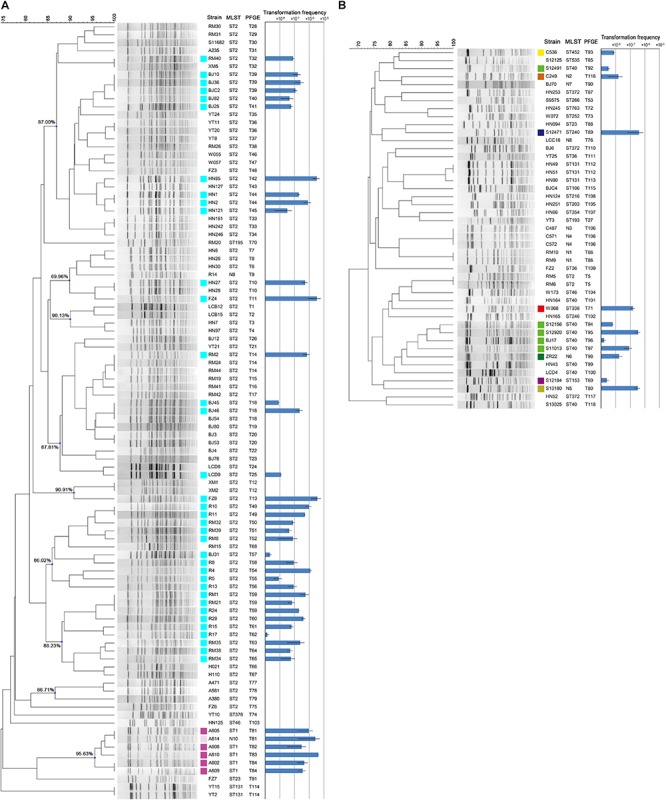
**(A)** PFGE typing and transformation frequencies of the 97 MDR clinical *A. baumannii* strains. Nine clusters were designated and the similarity values were provided. **(B)** PFGE typing and transformation frequencies of the 45 sensitive clinical *A. baumannii* strains. Error bars represent ± standard deviation. The results of MLST are provided for direct comparison. Isolates became transformable are marked by colored squares, and the colors indicate the MLST STs of the isolates. ST2(2-2-2-2-2-2-2), ST1(1-1-1-1-5-1-1), N10(1-1-1-1-5-2-1), ST1 like, ST40(1-2-2-2-5-1-14), N6(1-2-7-2-5-1-14), ST40 like, ST452(7-2-2-1-8-4-4), ST153(38-1-14-3-12-1-5), N2(12-1-7-1-7-2-4), ST338(8-5-5-26-13-1-2), N5(3-2-7-1-7-1-4), and ST240(3-3-2-5-7-2-51).

The 142 *A. baumannii* isolates were clustered into 9 groups of related genotypes using PFGE (Figure 2). No correspondence between the genotypic cluster and transformation frequency was revealed, but 3 of the 9 clusters showed a higher transformation frequency than the rest (Figure 2). Some closely related strains showed similar transformation frequencies; for example, isolates shared genotype T39. However, there were also instances in which closely related isolates varied markedly; for example, only two of the three T18 isolates were transformable, and the frequency varied by two orders of magnitude (Figure 2).

Among the strains studied here, a total of 97 MDR and 45 sensitive isolates were identified. The MDR *A. baumannii* strains were typed into 9 STs and 78 PFGE types, respectively. By contrast, the 45 sensitive strains show higher genetic diversity, which were typed into 29 STs and 40 PFGE types (Figure 2). Using the planktonic salt-free transformation assay, 38% (54/142) of the clinical *A. baumannii* isolates were natural competent. Although there was no significant difference in transformation rate between the MDR and sensitive strains (43.3%, 42/97 vs. 26.7%, 12/45, chi-square test, *P* = 0.0575), their transformation frequencies was significantly different. The transformation frequencies varied by up to four orders of magnitude, between 1.39 × 10^–8^ and 4.07 × 10^–5^ (Figure 2). Including all the transformation negative isolates, the MDR isolates showed higher transformation frequencies (mean ± SD, 2.75 × 10^–6^ ± 7.63 × 10^–6^) than the sensitive isolates (mean ± SD, 2.98 × 10^–7^ ± 9.1 × 10^–7^) (Mann-Whitney test, *P* = 0.015, Figure 2). All the transformable MDR isolates belong to ICL2 (ST2) or ICL1 complex (ST1 and N10), but no significant difference was revealed between them. A total of 34 STs were revealed in our study, and the 54 transformable isolates belonged to 11 of them (Table 3).

**TABLE 3:** Transformation frequencies in an MLST sequence type subset of *A. baumannii* strains.

**ST^*a*^**	**Allelic profile**	**No. of isolates**	**No. of transformable**	**Drug resistance**	**Mean transformation frequency (range)**
N3	12-3-16-1-13-1-16	1	0	S	Not detected
N7	4-1-2-3-12-1-5	1	0	S	Not detected
N9	2-2-2-2-2-S2-2	1	0	R	Not detected
N8	7-128-S2-1-8-1-4	1	0	S	Not detected
ST106	3-3-16-1-13-1-1	1	0	S	Not detected
ST193	3-1-7-1-7-2-4	1	0	S	Not detected
ST195	2-2-105-2-2-1-2	1	0	R	Not detected
ST203	3-4-2-2-7-1-2	1	0	S	Not detected
ST216	1-4-2-2-7-1-2	1	0	S	Not detected
ST246	1-49-3-4-5-2-36	1	0	S	Not detected
ST252	1-4-3-2-9-1-5	1	0	S	Not detected
ST266	27-2-2-2-7-1-5	1	0	S	Not detected
ST354	3-2-2-2-7-1-7	1	0	S	Not detected
ST376	27-4-2-1-42-1-2	1	0	R	Not detected
ST535	3-3-6-2-28-4-4	1	0	S	Not detected
ST763	3-4-2-2-9-1-5	1	0	S	Not detected
N1	147-2-2-1-151-1-3	2	0	S	Not detected
ST23	1-3-10-1-4-4-4	2	0	S/R	Not detected
ST36	1-2-2-2-3-1-2	2	0	S	Not detected
ST46	5-12-11-2-14-9-14	2	0	S/R	Not detected
N4	3-3-16-1-13-1-16	2	0	S	Not detected
ST372	1-4-2-1-42-1-2	3	0	S	Not detected
ST131	3-2-2-2-3-2-6	5	0	S/R	Not detected
N2	12-1-7-1-7-2-4	1	1	S	1.53 × 10^–7^
N5	3-2-7-1-7-1-4	1	1	S	3.22 × 10^–6^
ST153	38-1-14-3-12-1-5	1	1	S	2.50 × 10^–8^
ST240	3-3-2-5-7-2-51	1	1	S	3.94 × 10^–6^
ST338	8-5-5-26-13-1-2	1	1	S	1.48 × 10^–6^
ST452	7-2-2-1-8-4-4	1	1	S	7.54 × 10^–8^
N6	1-2-7-2-5-1-14	1	1	S	1.69 × 10^–7^
ST40	1-2-2-2-5-1-14	9	5	S	8.68 × 10^–7^ (1.6 × 10^–8^∼3.4 × 10^–6^)
N10	1-1-1-1-5-2-1	1	1	R	2.59 × 10^–5^
ST1	1-1-1-1-5-1-1	5	5	R	1.22 × 10^–5^ (3.04 × 10^–6^∼4.07 × 10^–5^)
ST2	2-2-2-2-2-2-2	86	36	R/S	4.98 × 10^–6^ (1.39 × 10^–8^∼3.59 × 10^–5^)
SUM		142	54		

*^*a*^New STs revealed in this study were named with Arabic numerals beginning with the letter N.*

### Competence Induction Time

**FIGURE 3:**
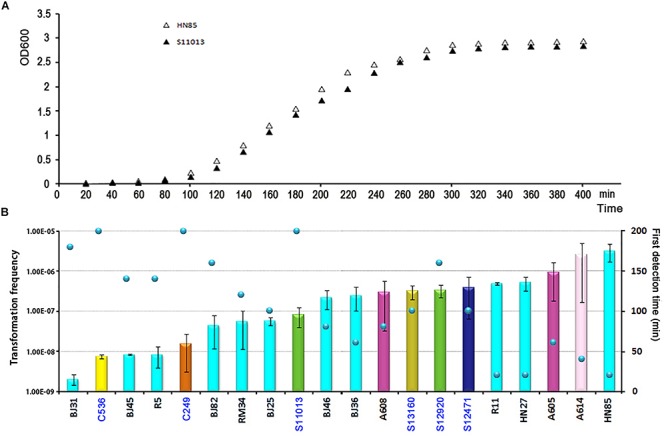
The competence induction time of *A. baumannii* isolates with different transformation frequencies. **(A)** Growth curve of the MDR *A. baumannii* isolate HN85 and the sensitive isolate S12920 in LB-no salt and LB, respectively. **(B)** The competence induction time of 20 *A. baumannii* isolates. The bar chart shows the transformation frequency, the color of the bar indicate the STs of the isolates, as shown in Figure 2. The sensitive strains were marked by blue fonts. The scatter plot shows the time point at which transformant colonies were first obtained.

To determine the timing of competence development during transformation, we followed this process by detecting transformation every 20 min. A total of 20 isolates covering 8 STs were selected. Growth experiments were performed for 2 isolates (HN85 and S11013) under the same culture conditions as the transformation assay by analyzing the optical density at 600 nm (OD600) at the time points indicated in Figure 3A. The time point that first showed transformant colonies varied depending on isolates, ranging from the first (20 min) to the tenth (200 min) detection time point, which covered from the lag phase to the middle of the exponential phase of *A. baumannii* growth (Figure 3A). Surprisingly, all the 11 tested ST2 isolates triggered competence much earlier than the sensitive isolates with a similar transformation frequency (Figure 3B). For example, the ST2 isolates R11 and HN27, which have a mean transformation frequency of 4.98 × 10^–6^ and 5.25 × 10^–6^, respectively, obtained successful transformation at the first detection time point (20 min), whereas the non-ST2 sensitive isolates S12920 and S12471, with transformation frequencies of 3.4 × 10^–6^ and 3.94 × 10^–6^, respectively, did not display transformation clones until 160 and 100 min, respectively.

## Discussion

The capacity to undergo natural competence has been studied for many bacterial species. For *Acinetobacter* spp., using *A. baylyi* strain ADP1, it was observed that an increase in nutrients plays a role in competence induction (Palmen et al., 1993; Porstendörfer et al., 2000). Here, we identified that subculturing with a high dilution factor into no/low salt broth could trigger competence for *A. baumannii*, under which conditions, 38% of the 142 clinical *A. baumannii* isolates were transformable, with transformation frequencies varied between 1.39 × 10^–8^ and 4.07 × 10^–5^. These data were similar to those obtained by the semi-solid media transformation method performed for kanamycin-sensitive clinical isolates (36% transformable, 11 out of 28, 3.91 × 10^–8^ 4.53 × 10^–6^) (Wilharm et al., 2013), but was lower than the semi-solid media transformation detected by fluorescence-based method (66% transformable, 8 out of 12, 10^–6^ 10^–2^) (Godeux et al., 2018). Compared with the semi-solid method, the novel planktonic salt-free transformation method is more simple and suitable to investigate the kinetics of *A. baumannii* DNA uptake.

Natural transformation is a multistep process (Chen and Dubnau, 2004). In many bacteria, the uptake of extracellular DNA is achieved by binding of the DNA to type IV pili (TFP) assembled at the surface of the recipient cell (Dubnau, 1999; Averhoff and Friedrich, 2003; Pelicic, 2008). Although the molecular mechanisms of *A. baumannii* DNA uptake remains unknown, the major pilin of TFP was reported to be necessary for *A. baumannii* natural transformation (Harding et al., 2013). So we speculate that the electrostatic interaction of the charged TFP surface with DNA might play a role. The charged ions produced by dissolution of inorganic salts may neutralize the charge of the DNA and/or TFP surface, thereby weakening their interaction. Thus, NaCl, as well as other inorganic salts tested in this study, could reduce the transformation frequency, and the decreased frequency was related to the concentration of NaCl. Osmolarity induced by sucrose did not exert any effect on the level of natural transformation, which supports our hypothesis that it is by affecting charge rather than osmolarity that NaCl interferes with DNA uptake.

Variation of temperature and NaCl concentration was reported to have no effect on *A. baumannii* A118, which is the first reported natural competent strain of *A. baumannii* (Maria Soledad et al., 2010; Traglia et al., 2016). However, in our research, 42°C and high NaCl concentrations significantly reduced the transformation frequency of *A. baumannii*. We speculate that A118 may have special DNA-uptake machinery that can trigger and operate differently with the strains studied herein because almost all of our transformable isolates failed to gain DNA uptake by the A118 transformation assay (late-stationary-death-phase culture diluted 1:1 with fresh LB broth), even when the LB broth was changed to LB-no salt broth. In addition, a recent study reported a similar effect of NaCl on *A. baumannii* A118, but the authors concluded that it was due to osmolarity changes, which is inconsistent with our results (Domingues et al., 2019).

The natural habitat of *A. baumannii* is not clearly defined, but the main characteristic of *A. baumannii* is the capacity to remain in the hospital environment on various inanimate materials, paving the way for the infection of vulnerable patients (Towner, 2009). No/low salt is not an extreme condition for the hospital environment, and the DNA may also be abundant in the hospital environment, so it can’t be ruled out that the competence triggered in laboratory can also occur in hospital environment.

Significant variation in transformation frequency between isolates is also observed in other bacterial species (Wilson et al., 2003; Maughan and Redfield, 2009; Evans and Rozen, 2013). It therefore appears that the variation we observe is a general feature of naturally transformable bacterial species. Although it is widely accepted that natural competence can contribute to bacterial evolution and adaptation to new niches, it also has several potential costs, from the metabolic expense of transcribing the newly acquired genetic material to the risk of importing deleterious alleles (Blokesch, 2017). The recipient cells should experience and balance these costs and benefits of transformation, so transformation frequency could increase or decrease rapidly. Therefore, the similar or distinct transformability variations among genotype-related isolates maintain themselves at their local fitness optimum, and new transformation frequencies can evolve over short evolutionary timescales.

*Acinetobacter baylyi* ADP1, the most well-studied transformable species in *Acinetobacter*, is always transformable during planktonic growth, though with large variations in efficiency throughout the growth cycle (Cruze et al., 1979). In contrast, our *A. baumannii* isolates showed variation in the competence induction time. Some ST2 isolates entered competence soon after inoculation during planktonic growth, so the DNA machinery may already be present in the late stationary growth phase of these isolates, diluting the cells into fresh medium providing the required energy allowed efficient DNA uptake and consequent transformation. However, some other isolates needed much longer time and did not trigger competence until the culture had reached the middle of the exponential growth phase, so these isolates may trigger competence only when reaching a high cell density. If this hypothesis is true, the *A. baumannii* transformation mechanism is more complicated than we anticipated, perhaps more than one set of DNA-uptake machinery and/or regulatory pathways could be utilized by different *A. baumannii* isolates, but these speculations need to be experimentally addressed in more detail.

Following previous work describing the acquisition of antibiotic resistance through natural transformation, we predicted that strains with higher transformation frequencies would exhibit greater levels of antibiotic resistance. However, we found that this was not the case. The sensitive isolates S12471 and S12920, for example, showed the same or even higher transformation frequency compared with some MDR isolates (Figure 2). The major defect for the sensitive isolates was that they trigger competence much later than the ICL2 isolates with a similar transformation frequency (Figure 3B). Therefore, we speculate that the competence trigger might be more important than transformation frequency in determining the success of *A. baumannii* transformation in nature. Competence induction time for the tested 6 sensitive isolates (transformation frequency, 7.54 × 10^–8^ ∼3.94 × 10^–6^), ranged from 100 min to 200 min, and the OD600 had reached 0.5 to 2.0 revealed by the growth curve (Figure 3A). If they require such a high cell density or reach exponential growth phase to start expressing competence genes, it will be difficult for them to obtain successful natural transformation in their natural habitat. As a contrast, the tested ST2 strains with similar transformation efficiency (8.39 × 10^–8^–5.25 × 10^–6^) had a competence trigger time of 20–160 min, which was shorter than the sensitive strains. If they really open competence earlier than the sensitive strains in nature, inevitably, it will give them more opportunities to obtain successful transformation and develop drug resistance.

The strains used in this study were isolated from clinical practice, so the ICL2 strains which were widely distributed in China occupied a large proportion. This is a limitation of this study that not so many isolates were selected for the other STs, so it’s difficult to define the natural transformation ability of other clones. But compared with strains with similar transformation frequency, the tested ST2 isolates induce competence faster. This may give them more opportunity to gain successful transformation in nature. This provides us an important clue to explain the severe drug resistance and clinical successfulness of ICL2. Although transformation was not detectable in 58% of the ST2 isolates, we could not exclude the possibility that the ancestral ST2 *A. baumannii* was naturally competent with outstanding transformation ability (fast and high) to rapidly acquire competitive advantages such as resistance genes and be disseminated. During the subsequent evolutionary process, variations in transformation frequency and the competence induction also accumulated, the non-competent and/or low competence posterity lineages were not efficiently eliminated or may have been actively selected for their fitness. This hypothesis refreshes our understanding of the resistance development of *A. baumannii* due to the distinct variations in DNA uptake ability between strains.

## Data Availability

All datasets generated for this study are included in the manuscript/supplementary files.

## Author Contributions

JZ and YH conceived and designed the experiments, and drafted the manuscript. YH, LH, XT, and FM performed the experiments. All authors read and approved the final manuscript.

## Conflict of Interest Statement

The authors declare that the research was conducted in the absence of any commercial or financial relationships that could be construed as a potential conflict of interest.

## Footnotes

1 http://pubmlst.org/abaumannii/
